# Vitamin D Status Does Not Affect Disability Progression of Patients with Multiple Sclerosis over Three Year Follow-Up

**DOI:** 10.1371/journal.pone.0156122

**Published:** 2016-06-08

**Authors:** Anne-Hilde Muris, Joost Smolders, Linda Rolf, Lieke J. J. Klinkenberg, Noreen van der Linden, Steven Meex, Jan Damoiseaux, Raymond Hupperts

**Affiliations:** 1 School for Mental Health and Neuroscience, Maastricht University Medical Center, Maastricht, the Netherlands; 2 Academic MS Center Limburg, Zuyderland Medical Center, Sittard, the Netherlands; 3 Central Diagnostic Laboratory, Maastricht University Medical Center, Maastricht, the Netherlands; University of Oxford, UNITED KINGDOM

## Abstract

**Background and Objective:**

The risk of developing multiple sclerosis (MS) as well as MS disease activity is associated with vitamin D (25(OH)D) status. The relationship between the main functional disability hallmark of MS, disability progression, and 25(OH)D status is less well established though, especially not in MS patients with progressive disease.

**Methods:**

This retrospective follow-up study included 554 MS patients with a serum baseline 25(OH)D level and Expanded Disability Status Scale (EDSS) with a minimum follow-up of three years. Logistic regressions were performed to assess the effect of baseline 25(OH)D status on relapse rate. Repeated measures linear regression analyses were performed to assess the effect on disability and disability progression.

**Results:**

Baseline deseasonalized 25(OH)D status was associated with subsequent relapse risk (yes/no), but only in the younger MS patients (≤ 37.5 years; OR = 0.872, per 10 nmol/L 25(OH)D, p = 0.041). Baseline 25(OH)D status was not significantly associated with either disability or disability progression, irrespective of MS phenotype.

**Conclusion:**

Within the physiological range, 25(OH)D status is just significantly associated with the occurrence of relapses in younger MS patients, but is not associated with disability or disability progression over three years follow-up. Whether high dose supplementation to supra physiological 25(OH)D levels prevents disability progression in MS should become clear from long term follow-up of supplementation studies.

## Introduction

Multiple sclerosis (MS), a demyelinating disease of the central nervous system (CNS), is considered to be an inflammatory disease of autoimmune origin.[1] A low vitamin D status, measured as 25(OH)D, has been associated with an increased risk of developing MS [2, 3] and vitamin D related genes, such as CYP24A1 and CYP27B1 emerge from gene wide association studies.[4]

Vitamin D status has also been associated with disease activity i.e. relapse rate. Firstly, MS patients showed lower vitamin D status during relapse than during remission.[5–7] Secondly, in early relapsing remitting MS (RRMS), higher 25(OH)D levels were associated with an increased chance of being relapse free 24 months prior to serum sampling [8], and with a decrease in the relapse rate in subsequent months by 9–34% with each 10 nmol/L increase in 25(OH)D level.[9–11] Some, but not all, small high dose supplementation studies showed an increase in the proportion of relapse-free patients [12], a lower-than-expected relapse rate [13], and a decrease in the number of gadolinium enhancing lesions on MRI [14, 15]. Ongoing randomized, placebo controlled trials on high-dose vitamin D supplementation will clarify its effect on relapse rate. [16]

At present, it is uncertain whether vitamin D is associated with the main functional disability hallmark in MS, disability progression. Cross-sectional studies have shown a negative correlation between 25(OH)D level and disability, yet causality is uncertain.[8, 17, 18] Higher sunlight exposure, being the main source of vitamin D, has been associated with a decreased likelihood of reaching milestones on the Expanded Disability Status Scale (EDSS) in RRMS and also with a decreased risk ratio of reaching milestones on the patient determined disability scale (PDSS) in progressive MS. [19, 20] Conversely, no correlation between 25(OH)D levels and the MS severity scale (MSSS) or EDSS has been found in African Americans [21] and the negative correlation between recent EDSS progression and 25(OH)D levels has not been retained after correcting for baseline EDSS in a prospective Tasmanian study.[17] Small, high dose vitamin D supplementation studies have revealed a reduced proportion of EDSS progressive RRMS patients [12] and a trend towards reduced disability [15]. In interferon β (IFNβ) treated or naive clinically isolated syndrome (CIS) patients, higher 25(OH)D levels, measured in the first months after diagnosis, predicted reduced disease activity and a slower rate of progression.[22]

The reduction of relapse risk associated with higher levels of 25(OH)D in the early, inflammatory course of the disease may be accompanied by a reduction of disability progression. [23, 24] Whether disability progression later on, may be predicted by vitamin D status is at present less clear. The answer to this is of great importance, since people with long standing MS constitute the largest proportion of patients and that number also includes the patients with progressive disease, who have the lowest 25(OH)D levels. [8] A low 25(OH)D status could be a target for intervention in these patients who have at present limited (RRMS) to no (progressive MS) therapeutic possibilities.

To elucidate further the effect of vitamin D status on disability progression, we conducted a retrospective, three-year follow-up study in which we assessed the predictive value of baseline 25(OH)D levels on relapse risk, EDSS disability, and EDSS progression in 554 MS patients. We show that 25(OH)D status, within the physiological range, is just significantly associated with the occurrence of relapses in younger MS patients, but is not associated with disability or disability progression over the three years follow-up.

## Methods

### Patients and study outcome measurements

All patients with MS according to the original or 2005 revised McDonald criteria [25, 26] were eligible for inclusion in the cohort. They all visited the Academic MS Center Limburg, the Netherlands in the period 2005 to 2013. This center was based at the Maastricht University Medical Center, Maastricht and is currently at the Zuyderland Medical Center, Sittard. Both cities are located in the south of the Netherlands at 51° latitude north. All patients visited the outpatient clinic for normal clinical care and were included when they had had a three year follow-up in EDSS [27] by the end of 2013, after a baseline serum 25(OH)D level measurement with corresponding EDSS within 6 months. During the visits to the outpatient clinic, relevant events regarding MS activity and progression were routinely registered. Clinical characteristics were recorded in all subjects according to the Dutch law on Medical Treatment Act (WGBO), the Personal Data Protection Act (Wbp) and the Code of Conduct for Health Research (Federa) (**S1 File**). [28] All data were anonymized before analysis. Data included (**S2 File**) age, sex, MS debut (date), MS phenotype [29] (RRMS, SPMS, PPMS; RRMS and SPMS patients were classified as RRMS-onset), date of MS diagnosis, start of progressive disease (date) if applicable, relapses (number and date), baseline serum 25(OH)D levels (nmol/L), baseline EDSS, at least 1 follow-up EDSS more than 3 years after inclusion, information on 25(OH)D supplementation and disease modifying treatments (DMTs). EDSS and the occurrence of relapses (defined as the development of new symptom(s) or aggravation of existing symptom(s) for at least 24 hours in a patient with stationary or improving disease course in the previous month [30]), were assessed by an experienced neurologist. Only EDSS during periods of remission (defined as having relapse free disease at least in the three months prior and half a month after measurement) were included. Patients who were included in high dose 25(OH)D supplementation (>800 IU, equal to >20μg) studies were excluded from the current study. Patients who were classified as having progressive relapsing MS were assigned to the SPMS group. The use of DMTs was highly variable between and within patients, because of treatment choices based on clinical patient care. Therefore, we were not able to include these data as an independent variable in our analysis.

Based on the inclusion criteria, 793 patients were deemed to be potential study subjects and their patient files were studied in detail. Eventually, 554 MS patients were included in the study (**S1 Fig**).

### 25(OH)D measurement

During the study period, serum 25(OH)D levels were measured by routine clinical analysis. Before 2008 (n = 196 samples), both a chemiluminescence immunoassay (CLIA) (Nichols Institute Diagnostics, California, USA) and a radioimmunoassay (Immunodiagnostics Systems, Boldon, UK) were used, due to a change in clinical diagnostic operating procedures. Both methods had a good inter-assay correlation.[8] After 2008 (n = 358 samples), a CLIA (LIAISON® 25 OH Vitamin D TOTAL Assay, Diasorin, Saluggia, Italy) was used.

In accordance with other studies, 25(OH)D data were deseasonalized to corrrect for seasonal variation of 25(OH)D levels.[17, 31, 32] The total of all consecutive 25(OH)D levels from all patients were used to model seasonal variation in our cohort according to the sinusoidal model described by van der Mei *et al*.[17] yt = β0 + β1sin(2πt/365)+ β2cos(2πt/365), where yt denotes serum 25(OH)D concentration, t denotes the day of the year when the sample was collected, and βj (j = 0,1,2) are estimated regression coefficients. The adjusted 25(OH)D value was calculated by applying the deviation of an individual from the population mean at a time-point measured, on the population mean at T = 0. The seasonal corrected 25(OH)D levels are referred to as vitamin D status.

### Statistical analysis

SPSS software (SPSS Inc., version 20.0, Chigaco, USA) was used to analyse the effect of the deseasonalized 25(OH)D status at baseline as the primary predictor of relapses during the three year follow-up period in RRMS-onset patients. This association was assessed by using either a logistic (yes/no) or ordinal logistic regressions (0,1,2,3 or more relapses over follow-up). Covariates included in these models were age at baseline (years), sex (M/F), disease duration (years), MS-phenotype (RRMS, SPMS), number of relapses in the three years prior to baseline, and EDSS at baseline. To answer the question whether the effects were MS-phenotype dependent the second order interaction baseline deseasonalized 25(OH)D*MS-phenotype, was investigated. Furthermore, potential relevant interactions with the primary outcome measure relapses, that were investigated were sex*age, sex*disease duration, age at baseline*baseline deseasonalized 25(OH)D, baseline deseasonalized 25(OH)D*baseline EDSS and the interactions with pre-study relapse rate. Interactions that were not significantly relevant for the model and did not investigate our primary research question were omitted from the final analyses using stepwise backward modelling.

Repeated measures linear regression analyses were performed to investigate the role of baseline deseasonalized 25(OH)D in EDSS progression and to account for the correlation between repeated measurements within the same patients. Based on the best fit of the model to our data (-2LL (REML) and BIC), a model with Toeplitz covariance structure was used to model these correlations. The robustness of the final model was verified by applying different covariance structures. The outcome measure EDSS was analysed as a continuous variable [33] and was measured over equal spaces in time (trimesters). All EDSS data between baseline and three year follow-up were included in the analyses. Besides baseline deseasonalized 25(OH)D status, other covariates of potential interest that were included in the model were follow-up time (trimesters), age at baseline (years), sex (M/F), disease duration (years), baseline EDSS, and MS-phenotype (RRMS, SPMS, PPMS). To look at the effects on EDSS change, all first order interactions with time were investigated. Other relevant interactions that may effect the role of deseasonalized 25(OH)D levels on disease progression, were included: disease duration*sex, baseline deseasonalized 25(OH)D*disease duration, baseline deseasonalized 25(OH)D*MS-phenotype, baseline EDSS*relapse rate pre study, age at baseline*relapse rate pre study, disease duration*relapse rate pre study, sex*relapse rate pre study and baseline EDSS*sex. Finally we also included the three way interaction baseline deseasonalized 25(OH)D*time*MS-phenotype, to investigate the MS-phenotype dependent effects of vitamin D on EDSS change. Interactions that were not significantly relevant for the model and did not investigate the relation between 25(OH)D and EDSS or EDSS progression were omitted from the analyses.

All of our tests were hypothesis driven. Three main outcome variables were tested: the relation of 25(OH)D status with relapses in three years follow-up, the relation with EDSS and with EDSS progression. Therefore the number of tests was minimal and correction for multiple testing was not applicable. A p-value of <0.05 was considered statistically significant.

## Results

### Patient characteristics and deseasonalization of 25(OH)D levels

**Fig 1:**
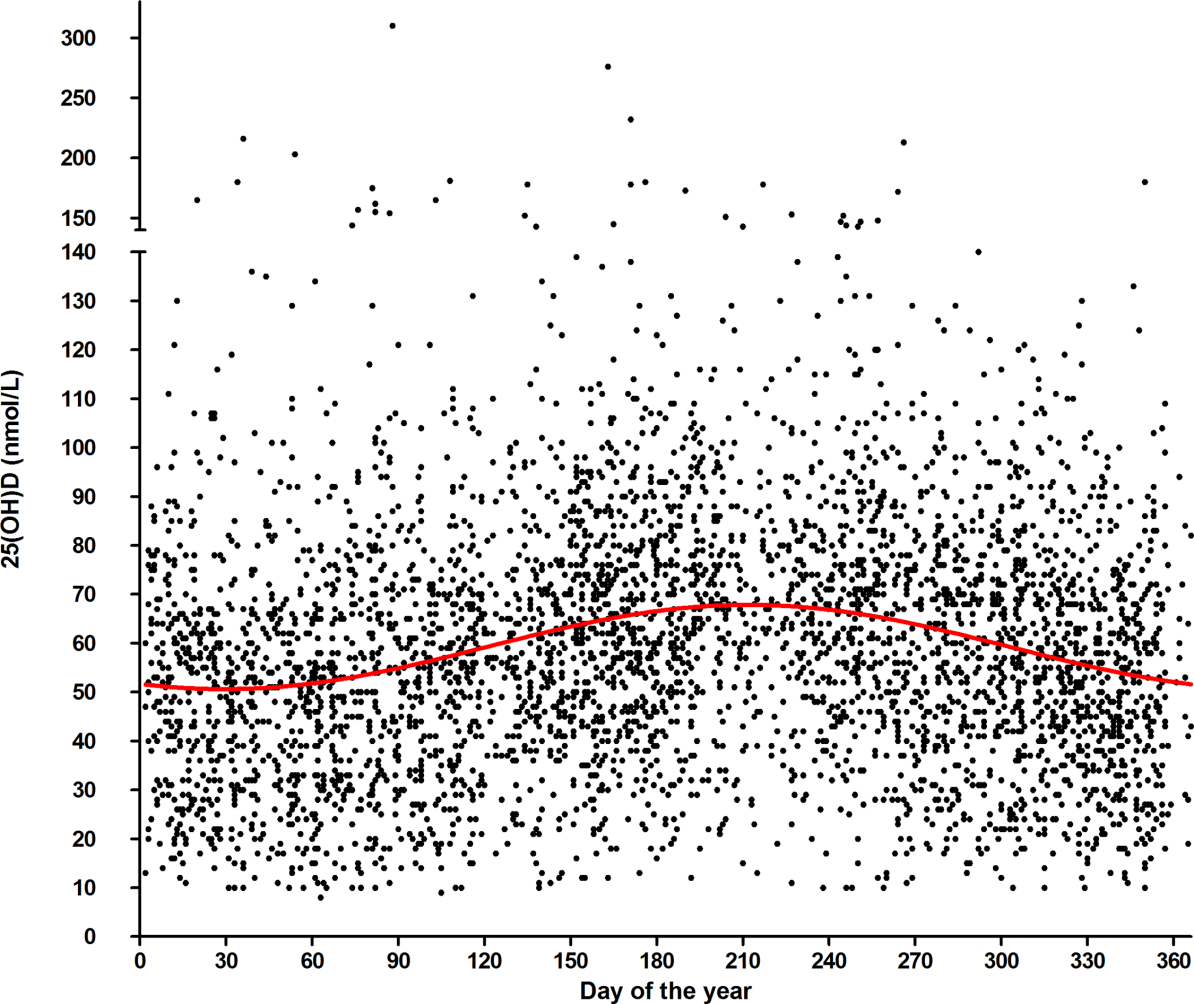
Seasonal fluctuation in 25(OH)D levels measured in the total Academic MS Center Limburg MS population (n = 4294 25(OH)D measurements). Each dot represents a 25(OH)D measurement of an MS patient. Formula of curve: 25(OH)D = 59.23–4.168*sin(2πt/365))—7.546*cos(2πt/365)).

The total study cohort comprised 554 patients. Patient characteristics are shown in **Table 1**. The characteristics of this selected study population did not differ from the total MS population at the Academic MS Center Limburg with respect to age at diagnosis, female/male ratio and MS-phenotype distribution (age at diagnosis 39.3±10.9 year; 71.6% female, 28.4% male; distribution of MS phenotype 51.8% RRMS, 34.1% SPMS and 14.0% PPMS). The model of the seasonal fluctuation of the 25(OH)D levels of the whole population had a mean of 59.2 nmol/L and an average fluctuation between 50.6 nmol/L and 67.9 nmol/L. Based on this data, the following formula was used for the deseasonalisation of all individual 25(OH)D data: deseasonalized 25(OH)D = crude 25(OH)D -(-4.168*sin(2πt/365))-7.546*cos(2πt/365)) (**Fig 1**).

**Table 1:** Characteristics of the selected study population of MS patients of the Academic MS Center Limburg. Data are provided as mean (SD) and as ^#^median (Q1-Q3) in case of skewed distributions.

*Population*		*Number*	*Percentage*
Number of patients		554	
Age (years)		46.7 (11.6)	
Sex (F/M)		395/158	71%/29%
MS phenotype			
	RRMS	340	61.4%
	SPMS	152	27.4%
	PPMS	62	11.2%
Disease duration (years)			
	since onset	12.5 (10.1)	
		^#^9.7 (4.2–18.8)	
	since diagnosis	7.2 (7.7)	
		^#^4.2 (0.9–11.3)	
Age (years)			
	at onset	34.2 (10.4)	
	at diagnosis	39.6 (10.8)	
Number of pre baseline relapses (in 3 years)			
	0	196	35.4%
	1	147	26.5%
	2	104	18.8%
	3 or more	107	19.3%
Baseline EDSS		4.0 (2.0–6.0)	
	≤3.5	274	49.5%
	4.0–5.5	129	23.2%
	≥6.0	151	27.3%
EDSS after 3 years follow-up		4.0 (2.5–6.5)	
	≤3.5	228	41.2%
	4.0–5.5	140	25.3%
	≥6.0	186	33.6%
Number of patients with EDSS progression after 3 years follow-up			
	<1	357	64.4%
	≥1	197	35.6%
Baseline 25(OH)D (nmol/L)			
	crude	57.5 (28.4)	
	deseasonalized	56.7 (28.0)	
Disease modifying treatment used most frequently during follow-up			
	none	225	40.6%
	first line treatment (interferon, glatiramer acetate)	193	34.8%
	second line treatment (natalizumab, fingolimod)	40	7.2%
	third line treatment (methotrexate, immunoglobulins, mitoxantrone)	75	13.5%
	Other	6	1.0%
	Unknown	15	2.7%
Number of relapses during three year follow-up			
	0	288	52.0%
	1	130	23.5%
	2	67	12.1%
	3 or more	69	12.5%

### Vitamin D status predicts the risk of relapses during three-year follow-up in younger RRMS-onset patients

In the RRMS-onset cohort (n = 490, characteristics in **S1 Table**), we studied the effect of baseline 25(OH)D level on relapse rate. Baseline deseasonalized 25(OH)D did not have an overall effect on the risk of getting relapses (yes/no) (OR = 0.96, CI OR = 0.89–1.03, p = 0.21, **Table 2**) or the number of relapses (**S2A and S2B Table**) in the follow-up period of three years. However, the effect of 25(OH)D status on the risk of getting relapses was age dependent (OR = 1.007, CI OR = 1.000–1.015, p = 0.047, **Table 3**), showing a low significant beneficial effect of deseasonalized baseline 25(OH)D status on relapses (yes/no) in RRMS-onset patients in the lowest age at baseline quartile ≤37.5 year (OR = 0.872, CI OR = 0.765–0.994, p = 0.041 in the most extensive model, **Table 4**), no effect in MS patients aged between 37.5 and 54 year, and a disadvantageous effect in the MS patients aged ≥54 year at baseline which was also just significant (OR = 1.430, CI OR = 1.003–2.040 p = 0.048, **Table 4**). Other predictors of relapses were age at baseline per se, EDSS at baseline and relapse rate pre study, the last factor being disease duration dependent.

**Table 2:** Association of vitamin D status and relapses (yes/no) during three year follow-up in RRMS-onset patients—Model 1: main variables.

Vitamin D and relapse risk in RRMS-onset population (logistic regression)				
*Parameter*	*B*	*Odds Ratio*	*95% CI OR*	*p-value*
**Relapses (ref = no)**				
Baseline 25(OH)D (per 10 nmol/L) deseasonalized	-0.045	0.956	0.892–1.025	0.206
SPMS (ref = RRMS)	-0.642	0.526	0.275–1.008	0.053
**Age at baseline (years)**	**-0.036**	**0.964**	**0.944–0.985**	**0.001**
Duration of disease (years)	0.024	1.024	0.994–1.055	0.112
Sex (ref. = female)	-0.183	0.833	0.538–1.290	0.413
**EDSS baseline**	**0.138**	**1.148**	**1.003–1.313**	**0.045**
**EDSS baseline**^**2**^ **(centered around mean of 3.8)**	**-0.093**	**0.912**	**0.869–0.956**	**<0.001**
**Relapse rate 3 years pre-baseline**	**0.243**	**1.275**	**1.107–1.467**	**0.001**

See for characteristics of the RRMS-onset study population S1 Table.

**Table 3:** Association of vitamin D status and relapses (yes/no) during three year follow-up in RRMS-onset patients—Model 2: model 1 plus interaction terms.

Vitamin D and relapse risk in RRMS-onset population (logistic regression)				
*Parameter*	*B*	*Odds Ratio*	*95% CI OR*	*p-value*
**Relapses (ref = no)**				
**Baseline 25(OH)D (per 10 nmol/L) deseasonalized**	**-0.333**	**0.717**	**0.530–0.970**	**0.031**
SPMS (ref = RRMS) *Baseline 25(OH)D (per 10 nmol/L) deseasonalized	-0.090	0.914	0.764–1.094	0.327
Age at baseline (years)*Baseline 25(OH)D (per 10 nmol/L) deseasonalized	**0.007**	**1.007**	**1.000–1.0145**	**0.047**
SPMS (ref = RRMS)	-0.222	0.801	0.248–2.592	0.712
**Age at baseline (years)**	**-0.077**	**0.926**	**0.884–0.970**	**0.001**
Duration of disease (years)	-0.010	0.990	0.951–1.030	0.610
Sex (ref. = female)	-0.217	0.805	0.516–1.257	0.340
**EDSS baseline**	**0.149**	**1.160**	**1.013–1.329**	**0.032**
**EDSS baseline**^**2**^ **(centered around mean of 3.8)**	**-0.093**	**0.911**	**0.868–0.957**	**<0.001**
Relapse rate 3 years pre-baseline	0.032	1.033	0.843–1.265	0.757

Corrected for “duration of disease (years)*relapse rate 3 years pre-baseline” B 0.029, OR 1.030, p = 0.012

See for characteristics of the RRMS-onset study population S1 Table.

**Table 4:** Association of vitamin D status and relapses (yes/no) during three year follow-up in RRMS-onset patients—per age categories (n = 123 per quartile) at baseline.

Vitamin D and relapse risk in RRMS-onset population (logistic regression)						
Effect of baseline 25(OH)D (per 10 nmol/L) deseasonalized						
*Age category*			*B*	*Odds Ratio*	*95% CI OR*	*p-value*
**Q1: <37.5 year**						
		**Simple model**	**-0.109**	**0.897**	**0.797–1.009**	**0.069**
		**Model 1**	**-0.120**	**0.890**	**0.786–1.007**	**0.065**
		**Model 2 (adjusted)**	**-0.137**	**0.872**	**0.765–0.994**	**0.041**
Q2: 37.5–46.2 year						
		Simple model	-0.065	0.937	0.824–1.066	0.323
		Model 1	-0.040	0.961	0.835–1.106	0.581
		Model 2 (adjusted)	-0.024	0.977	0.834–1.144	0.770
Q3: 46.2–54.0 year						
		Simple model	-0.056	0.946	0.836–1.070	0.376
		Model 1	-0.075	0.928	0.806–1.068	0.296
		Model 2 (adjusted)	0.013	1.013	0.855–1.200	0.883
**Q4: >54.0 year**						
	**Simple model**		**0.203**	**1.226**	**1.030–1.460**	**0.022**
	Model 1		0.140	1.151	0.943–1.404	0.166
	**Model 2 (adjusted)**		**0.358**	**1.430**	**1.003–2.040**	**0.048**

See for characteristics of the RRMS-onset study population S1 Table.**Simple model** includes only baseline 25(OH)D deseasonalized as covariate. **Model 1** includes baseline 25(OH)D deseasonalized, MS phenotype, age at baseline, duration of disease, sex, EDSS baseline, relapse rate three years pre-study, and EDSS baseline^2^ (centered) as covariates. **Model 2 (adjusted)** same as Model 1 but including MS phenotype*baseline 25(OH)D deseasonalized, and duration of disease*pre-study relapse rate as covariates and excluding age at baseline*baseline 25(OH)D deseasonalized

### Vitamin D status does not predict disability and disability progression in multiple sclerosis patients over three year follow-up

Baseline deseasonalized 25(OH)D status did not have any overall effect on EDSS (β = -0.003, p = 0.615 (**S3A Table**)) or EDSS change over three year follow up (β = -0.002, p = 0.790 (**Table 5**) in the simple model and β = -0.011, p = 0.113 in the more extensive model (**Table 6**)). Also after correction for MS-phenotype, 25(OH)D did not predict EDSS change over time (β = 0.021, p = 0.198 for SPMS and β = 0.013, p = 0.621 for PPMS with RRMS as reference, **S3B Table**) which was confirmed by an MS phenotype subgroup analyses (data not shown). MS phenotype at baseline, age at baseline, EDSS at baseline and relapse rate three years pre-baseline were the most important predictors in the model for EDSS change over time (**Table 6**). In addition, whether patients had stable or progressive disease (≥1 EDSS increase over three year follow-up) was not dependent on baseline 25(OH)D status in a logistic regression model (OR = 1.021, CI OR = 0.952–1.095, p = 0.560).

**Table 5:** Effect of vitamin D status on EDSS over 3 year follow-up according to repeated measures linear regression analyses with Toeplitz covariance structure—Model 1: main variables.

Repeated measures linear regression analyses of longitudinal EDSS data with Toeplitz covariance structure				
*Parameter*		*β*	*95% CI β*	*p-value*
Baseline 25(OH)D (per 10 nmol/L) deseasonalized		-0.002	-0.014–0.0100	0.733
Baseline 25 (OH)D deseasonalized *time since baseline (per 10 nmol/L per year)		-0.002	-0.016–0.012	0.790
MS phenotype at baseline (ref. = RRMS)				
	SPMS	0.049	-0.054–0.152	0.353
	PPMS	0.070	-0.039–0.180	0.208
**Time since baseline (years)**		**0.156**	**0.072–0.244**	**<0.001**
Age at baseline (years)		0.002	-0.001–0.006	0.148
Duration of disease (years)		0.001	-0.004–0.006	0.809
Sex (ref. = female)		0.015	-0.053–0.082	0.670
**EDSS baseline**		**0.962**	**0.941–0.983**	**<0.001**
EDSS baseline*EDSS baseline (centered around mean of 3.8)		**0.010**	**0.002–0.017**	**0.012**
Relapse rate 3 years pre-baseline		0.005	-0.017–0.026	0.679

**Table 6:** Effect of vitamin D status on EDSS over 3 year follow-up according to repeated measures linear regression analyses with Toeplitz covariance structure—Model 2: Model 1 plus two way interaction terms.

Repeated measures linear regression analyses of longitudinal EDSS data with Toeplitz covariance structure				
*Parameter*		*β*	*95% CI β*	*p-value*
Baseline 25(OH)D (per 10 nmol/L) deseasonalized		-0.0037	-0.017–0.010	0.595
Baseline 25(OH)D deseasonalized*time since baseline (per 10 nmol/L*year)		-0.011	-0.024–0.003	0.113
MS phenotype at baseline (ref. = RRMS)*Baseline 25(OH)D (per 10 nmol/L) deseasonalized				
	SPMS	0.017	-0.008–0.043	0.175
	PPMS	0.014	-0.025–0.053	0.479
MS phenotype at baseline (ref. = RRMS)				
	SPMS	-0.132	-0.307–0.043	0.139
	PPMS	-0.087	-0.330–0.155	0.480
**Time since baseline (years)**		**0.415**	**0.208–0.623**	**<0.001**
Age at baseline (years)		0.0004	-0.0031–0.0039	0.831
Duration of disease (years)		0.003	-0.002–0.008	0.285
**Sex (ref. = female)**		**0.092**	**0.001–0.183**	**0.048**
**EDSS baseline**		**1.012**	**0.989–1.035**	**<0.001**
**EDSS baseline**^**2**^ **(centered around mean of 3.8)**		**0.011**	**0.003–0.018**	**0.004**
Relapse rate 3 years pre-baseline		0.016	-0.007–0.040	0.173
MS phenotype at baseline (ref. = RRMS)*time since baseline (years)				
	**SPMS**	**0.237**	**0.119–0.355**	**<0.001**
	**PPMS**	**0.170**	**0.043–0.297**	**0.009**
Age at baseline (years)*time since baseline (years)		**0.005**	**0.002–0.009**	**0.006**
EDSS baseline*time since baseline (years)		**-0.130**	**-0.154–0.106**	**<0.001**
Relapse rate 3 years pre-baseline*time since baseline (trimesters)		**-0.031**	**-0.058–0.004**	**0.023**

Corrected for duration of disease (years)*sex B -0.010, p = 0.029

## Discussion

In this study, we retrospectively assessed the relation between baseline 25(OH)D status and EDSS progression over three year in an unselected, real life, clinical MS population. Disease disability per se, as expressed by EDSS, was not affected by baseline 25(OH)D levels and also the accumulation of disability over time was not correlated with baseline 25(OH)D status. In our population there was a strong trend towards 25(OH)D status predicting the occurrence of relapses, but only in MS patients in the youngest and the oldest age groups.

There are several mechanisms which support the hypothesis that a higher vitamin D status may reduce disability progression both in early MS-patients with inflammatory disease and later on in established or progressive MS. First of all by the proposed immunomodulatory effects of vitamin D [34], which could prevent relapses in RRMS-onset patients, thereby preventing accumulation of tissue damage. It has been shown that recovery, post relapse, will be incomplete in a substantial proportion of about 50% of the patients, leading to an increased EDSS.[23] In the present study, we indeed showed that vitamin D status most probably can predict the occurrence of relapses in younger MS patients. Furthermore, vitamin D could have a direct beneficial effect on the brain in late MS, by either neuroprotective effects, maintaining the blood brain barrier integrity, decreasing axonal damage or improving remyelination.[35–37] Additionally, the vitamin D responsiveness of the CNS may be increased due to neuroinflammation.[37] Our data do not support these hypotheses, though. However, they do confirm the results found by van der Mei *et al*.[17] and Løken-Amsrud *et al*. [38] who were also unable to detect a significant association of vitamin D status with EDSS progression, whether corrected for baseline EDSS or not. Soilu-Hanninen *et al*.[15] found (a trend towards) a relation between 25(OH)D levels and EDSS in their supplementation study in which patients with supplementation had a mean 25(OH)D level of 110 (67–163) nmol/L after 12 months. However, our patient population did not receive high-dose supplementation, was larger, and the follow up was longer. Furthermore, while most vitamin D association studies in MS were performed in selected patient cohorts, we deliberately have chosen for an unselected, real life MS population, in order to obtain robust results that can be used in clinical practice. In contrast to what we found in our unselected, clinical MS patient population, Ascherio *et al*.[22] showed that in CIS patients a 50 nmol/L increase in 25(OH)D levels was associated with a reduction of 0.16 EDSS points per year. This change was lower in the highest vitamin D ranges. Altogether this may indicate that vitamin D indeed plays a role in the onset and disease activity in young, early MS patients with inflammatory disease, but not at a later stage in the progression of disability. It has been suggested that once a clinical threshold of 4 EDSS points is reached, relapses no longer have any effect on the progression of disability.[24, 39] This might explain why the 25(OH)D status in young MS patients is related to the occurrence of relapses but did not show any overall association with disability progression.

Although we could not demonstrate any effect of vitamin D status on EDSS progression, also established and often used treatments in MS do not have an immense effect on EDSS progression. First line medication, such as IFNβ, has been shown to decrease EDSS in RRMS by 0.7 points in high dose, and by 0.1 points in low doses in 24 months.[40] The pooled effect size in a meta-analysis was non-significant over 24 months and IFNβ was also not associated with a change in disability progression in a big retrospective cohort study with a minimum follow-up of 4 years.[41, 42] Also in SPMS, no significant differences on the EDSS change were found between low dose IFNβ1a and high dose IFNβ1a.[43] Glatiramer acetate was associated with an EDSS change of 0.6 (±2.0) over 15 years while withdrawn patients had a mean change of 1.0 (±1.7).[44] With natalizumab the mean EDSS-change was comparable for those patients who had ongoing treatment and the patients who withdrew (mean change 0.05 vs. 0.07). Over nearly 5 years, mean EDSS remained stable in both groups, while the difference between placebo and natalizumab remained significant (3.15 vs. 2.79).[45]

There are certain limitations in this study. First of all, this was a retrospective study and baseline 25(OH)D levels and EDSS were measured in a clinical setting, unrelated in timing to disease onset or diagnosis and not fixed to study visits. Second, it investigated the effect of 25(OH)D status in a, although clinically relevant and unselected, heterogeneous population, which might have led to a biased estimate of the true effect size. Third, the follow-up in this study was limited to a three year follow-up. Fourth, a majority of patients took low dose vitamin D supplementation (400/800 IU/day, equal to 10–20μg/day), which might have underestimated the outcomes. Vitamin D status showed a regression to the mean over three year follow up (data not shown), which has also been described earlier.[22, 46] Other than the effect of supplementation, this regression to the mean might be due to lifestyle changes of the MS patients. Other important co-factors such as BMI, smoking habits and the use of DMTs could not be included. The fact that 25(OH)D levels were measured by different assays before and after 2008, did not change the vitamin D effect on disease activity and disease progression as was tested by adding this factor as a covariate to the models. Finally, EDSS as an outcome measure is not the ultimate measure with significant intra- and interrate differences and a non-linear course. However, it is the only frequently used measure available to monitor disability.

While it was not possible to detect any effect of vitamin D status on disability progression, it might still be possible that (high dose) vitamin D supplementation is beneficial in MS patients with long standing and progressive disease. We examined 25(OH)D levels in the physiological range in patients taking only low doses of vitamin D supplementation. This might have underestimated the effect of 25(OH)D status on EDSS (progression), but it might also be that high dose supplementation is needed to exert its effects on the immune system and the brain. Secondly, supplementation might be beneficial particularly in patients with the lowest 25(OH)D status. Finally, we were not able to take into account all the treatments patients received and, therefore, could not investigate the possible add-on or synergistic effects that vitamin D might have, as has been suggested for IFNβ and fingolimod.[46–49] Noticeably, this presumed interaction between vitamin D and IFNβ could not be reproduced by others. [38, 50]

To date, evidence that vitamin D status affects disability progression in MS patients could not be found. So far, the small effects of vitamin D on disease activity and disability in MS seem to be mainly visible in well selected homogeneous subgroups of MS patients, which makes translation to the MS population in the daily clinical practice difficult. Long term follow-up studies of high-dose vitamin D supplementation have to confirm these results. Before these data are available, though, it might be worthwhile considering preventive vitamin D supplementation in young (CIS and early) MS patients.

## Supporting Information

S1 Fig Flow chart inclusion of study patients. (DOCX)

S1 File METC approval. (PDF)

S2 File Data set. (XLS)

S1 Table Characteristics of the RRMS-onset study population of MS patients of the Academic MS Center Limburg. (DOCX)

S2 Table Association of vitamin D status and relapses (0, 1, 2, 3 or more) during three year follow-up in RRMS-onset patients. (DOCX)

S3 Table Additional analyses of the effect of vitamin D status on EDSS over 3 year follow-up according to repeated measures linear regression with Toeplitz covariance structure. (DOCX)
