# Supplementary figures and images for: Vitamin D Status Does Not Affect Disability Progression of Patients with Multiple Sclerosis over Three Year Follow-Up
Source: PLoS One. 2016 Jun 8;11(6):e0156122. doi: 10.1371/journal.pone.0156122 (PMC4898831; doi:10.1371/journal.pone.0156122)

**S1** Flow chart inclusion of study patients

**
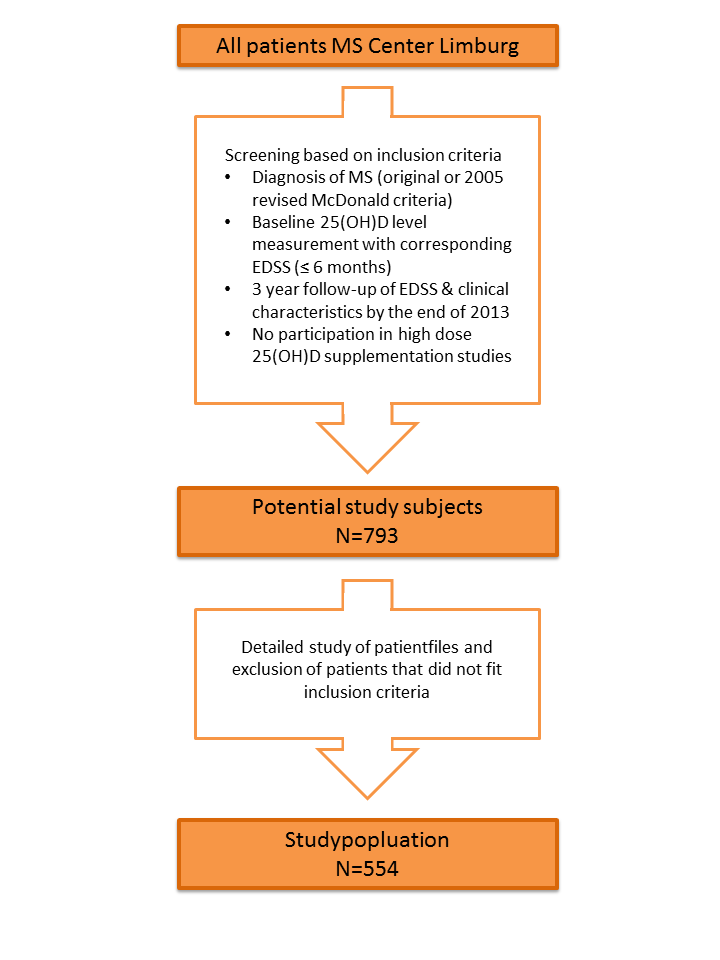
**

Supplement: S1 Fig — (DOCX) [file pone.0156122.s001.docx]
