# Supplementary material for: Vitamin D Status Does Not Affect Disability Progression of Patients with Multiple Sclerosis over Three Year Follow-Up
Source: PLoS One. 2016 Jun 8;11(6):e0156122. doi: 10.1371/journal.pone.0156122 (PMC4898831; doi:10.1371/journal.pone.0156122)
Supplement: S1 File — (PDF) [file pone.0156122.s002.pdf]

METC Z
Zuyderland - Zuyd

Mrs. Anne-Hilde Muris
School for Mental Health and Neuroscience
Maastricht Universitair Medisch Centrum
Universiteitssingel 40, kamer C2.558
Postbus 616
6200 MD Maastricht

**Date** February, 15, 2016
**CEC number** 16-N-45
**Title** 'Vitamin D Status Does Not Affect Disability Progression of Patients with Multiple Sclerosis over Three Year Follow-up'

The committee assessed the following documents received 14th of February 2016:

- Your letter from February, 14, 2016
- Protocol titled 'Vitamin D and disability progression in MS patients'
- CV A. Muris, February, 2016

Referring to your study it is hereby confirmed that the Medical Research Involving Human Subjects Act (WMO) does not apply to the above mentioned study and that therefore an official approval of this study by METC Z is not required under the WMO. Assuming the researchers work in respect with the Code of Good Conduct as well as the WBP (data protection act) we do not have any concerns against this study.

At the end of the study, the committee will receive a report and/or an article.

Kind regards,
On behalf of METC Z,
Dr. J.W. Greve
President

Mrs. mr. H. van den Besselaar, Secretary

copy: Zuyderland Board
a.muris@maastrichtuniversity.nl

The Research Ethics Committee (METC Z) of Zuyderland and Zuyd University tests, within the context of WMO medical-scientific research, including clinical research in the first-line, care and rehabilitation sector, in accordance with ICH-Good Clinical Practice (ICH-GCP), Medical Research Involving Human Subjects Acts (WMO) and the External Review Directive (RET). METC Z is recognized by the Central Committee on Research Involving Human Subjects (CCMO).

Correspondentieadres
METC Z
Secretariaat
Postbus 5500
6130 MB Sittard

metc@atriummc.nl

Bezoekadressen
Leerhuis Gebouw V
Heerlen (ma-di-wo-do)
H. Dunantstraat 5
T 045 – 5767 194

Leerhuis BWO K&E A
Sittard-Geleen (wo)
Dr. van der Hoffplein 1
T 088 - 459 7347

www.atriummc.nl/METC

METC Z leden:
dr. J.W. Greve (voorzitter)
dr. J. Kragten (vicevoorzitter)
mw. drs. L. Dielis
mw. mr. dr. R. ten Hoopen
dr. R. Janknegt
ir. drs. A. Kessels
dr. H. van der Kuy
mw. dr. A. Moser
mw. dr. B. Panis
dr. M. Reinders
drs. H. Rijksen
dr. S. Samijo
drs. B. Simons
drs. J. van der Snoek
mw. mr. L. Teeuwen
dr. A. Voogd

vaste adviseurs:
dr. W. van Asten
mw. dr. A. van den Hout

METC Z secretariaat:
mw. mr. H. van den Besselaar
mw. J. Jennekens
mw. G. Spreeuwenberg
mw. B. Westerkamp
