# Supplementary material for: Vitamin D Status Does Not Affect Disability Progression of Patients with Multiple Sclerosis over Three Year Follow-Up
Source: PLoS One. 2016 Jun 8;11(6):e0156122. doi: 10.1371/journal.pone.0156122 (PMC4898831; doi:10.1371/journal.pone.0156122)
Supplement: S1 Table — (DOCX) [file pone.0156122.s004.docx]

**S2 Characteristics of the RRMS-onset study population of MS patients of the Academic MS Center Limburg. Data are provided as mean (SD) and as ^#^median (Q1-Q3) in case of skewed distributions.**

| **Study *Population*** | | | ***N Number*** | ***Percentage*** |
| --- | --- | --- | --- | --- |
| Number of patients | | 492 | |  |
| Age (years) | | 45.9 (11.6) | |  |
| Sex (F/M) | | 362/129 | | 73.6%/26.2% |
| MS phenotype | |  | |  |
|  | RRMS | 340 | | 69.1% |
|  | SPMS | 152 | | 30.9% |
| Disease duration (years) | |  | |  |
|  | since onset | 12.7 (10.2) | |  |
|  |  | ^#^10.2 (4.2-19.2) | |  |
|  | since diagnosis | 7.5 (7.8) | |  |
|  |  | ^#^4.5 (1.1-11.8) | |  |
| Age (years) | |  | |  |
|  | at onset | 33.2 (10.0) | |  |
|  | at diagnosis | 38.4 (10.5) | |  |
| Number of pre baseline relapses during three year | |  | |  |
|  | 0 | 151 | | 30.7% |
|  | 1 | 138 | | 28.0% |
|  | 2 | 97 | | 19.7% |
|  | 3 or more | 106 | | 21.5% |
| Baseline EDSS | |  | |  |
|  | ≤3.5 | 254 | | 51.6% |
|  | 4.0-5.5 | 108 | | 22.0% |
|  | ≥6.0 | 130 | | 26.4% |
| Baseline 25(OH)D (nmol/L) | |  | |  |
|  | crude | 58.0 (28.9) | |  |
|  | deseasonalized | 57.4 (28.5) | |  |
| Number of post baseline relapses during three year | |  | |  |
|  | 0 | 232 | | 47.3% |
|  | 1 | 124 | | 25.3% |
|  | 2 | 67 | | 13.7% |
|  | 3 or more | 67 | | 13.7% |
