# Supplementary material for: Vitamin D Status Does Not Affect Disability Progression of Patients with Multiple Sclerosis over Three Year Follow-Up
Source: PLoS One. 2016 Jun 8;11(6):e0156122. doi: 10.1371/journal.pone.0156122 (PMC4898831; doi:10.1371/journal.pone.0156122)
Supplement: S2 Table — (DOCX) [file pone.0156122.s005.docx]

**S3 Association of vitamin D status and relapses (0, 1, 2, 3 or more) during three year follow-up in RRMS-onset patients**

**A Model 1: main variables**

| **Vitamin D and relapse risk in RRMS-onset population (ordinal logistic regression)** | | | | |
| --- | --- | --- | --- | --- |
| ***Parameter*** | ***β*** | ***Odds Ratio*** | ***95% CI OR*** | ***p-value*** |
| Baseline 25(OH)D (per 10 nmol/L) deseasonalized | -0.050 | 0.952 | 0.895-1.012 | 0.117 |
| **SPMS (ref = RRMS)** | **-0.728** | **0.483** | **0.269-0.867** | **0.015** |
| **Age at baseline (years)** | **-0.035** | **0.966** | **0.948-0.984** | **<0.001** |
| Duration of disease (years) | 0.017 | 1.017 | 0.991-1.045 | 0.201 |
| Sex (ref. = female) | -0.137 | 0.872 | 0.586-1.296 | 0.499 |
| **EDSS baseline** | **0.152** | **1.164** | **1.034-1.310** | **0.012** |
| **EDSS baseline^2^ (centered around mean of 3.8)** | **-0.078** | **0.925** | **0.886-0.967** | **0.001** |
| **Relapse rate 3 years pre-baseline** | **0.252** | **1.287** | **1.142-1.450** | **<0.001** |

*See for characteristics of the RRM- onset study population table S2 file.*

*Test of parallel lines: p=0.964*

**B Model 2: Model 1 plus interaction terms**

| **Vitamin D and relapse risk in RRMS-onset population (ordinal logistic regression)** | | | | |
| --- | --- | --- | --- | --- |
| ***Parameter*** | ***β*** | ***Odds Ratio*** | ***95% CI OR*** | ***p-value*** |
| Baseline 25(OH)D (per 10 nmol/L) deseasonalized | -0.041 | 0.960 | 0.895-1.029 | 0.249 |
| SPMS (ref = RRMS)*Baseline 25(OH)D (per 10 nmol/L) deseasonalized | -0.022 | 0.998 | 0.983-1.013 | 0.770 |
| SPMS (ref = RRMS) | -0.725 | 0.484 | 0.184-1.273 | 0.142 |
| **Age at baseline (years)** | **-0.034** | **0.966** | **0.948-0.985** | **<0.001** |
| Duration of disease (years) | -0.012 | 0.988 | 0.954-1.024 | 0.519 |
| Sex (ref. = female) | -0.136 | 0.872 | 0.586-1.300 | 0.502 |
| **EDSS baseline** | **0.164** | **1.178** | **1.046-1.327** | **0.007** |
| **EDSS baseline^2^ (centered around mean of 3.8)** | **-0.076** | **0.927** | **0.887-0.969** | **0.001** |
| Relapse rate 3 years pre-baseline | 0.093 | 1.097 | 0.924-1.304 | 0.290 |

*See for characteristics of the RRMS-onset study population table S2 file.*

*Test of parallel lines: p=0.925*

*Corrected for duration of disease (years)*relapse rate 3 years pre-baseline B 0.020, OR 1.020, p=0.017*
