# Supplementary material for: Vitamin D Status Does Not Affect Disability Progression of Patients with Multiple Sclerosis over Three Year Follow-Up
Source: PLoS One. 2016 Jun 8;11(6):e0156122. doi: 10.1371/journal.pone.0156122 (PMC4898831; doi:10.1371/journal.pone.0156122)
Supplement: S3 Table — (DOCX) [file pone.0156122.s006.docx]

**S4 Additional analyses of the effect of vitamin D status on EDSS over 3 year follow-up according to repeated measures linear regression analyses with Toeplitz covariance structure**

**A Effect on EDSS independent of time**

| **Repeated measures linear regression analyses of longitudinal EDSS data with Toeplitz covariance structure** | | | | |
| --- | --- | --- | --- | --- |
| ***Parameter*** | | ***β*** | ***95% CI β*** | ***p-value*** |
| Baseline 25(OH)D (per 10 nmol/L) deseasonalized | | -0.003 | -0.014-0.008 | 0.615 |
| MS phenotype at baseline (ref. = RRMS) | |  |  |  |
|  | SPMS | 0.049 | -0.054-0.152 | 0.353 |
|  | PPMS | 0.070 | -0.039-0.180 | 0.208 |
| **Time since baseline (years)** | | **0.147** | **0.109-0.185** | **<0.001** |
| Age at baseline (years) | | 0.002 | -0.001-0.006 | 0.148 |
| Duration of disease (years) | | 0.001 | -0.004-0.006 | 0.810 |
| Sex (ref. = female) | | 0.015 | -0.055-0.082 | 0.671 |
| **EDSS baseline** | | **0.962** | **0.941-0.983** | **<0.001** |
| **EDSS baseline^2^ (centered around mean of 3.8)** | | **0.097** | **0.002-0.017** | **0.012** |
| Relapse rate 3 years pre-baseline | | 0.005 | -0.017-0.026 | 0.677 |

**B Model 3: Model 2 (see table 6.) plus three way interaction terms**

| **Repeated measures linear regression analyses of longitudinal EDSS data with Toeplitz covariance structure** | | | | |
| --- | --- | --- | --- | --- |
| ***Parameter*** | | ***β*** | ***95% CI β*** | ***p-value*** |
| Baseline 25(OH)D (per 10 nmol/L) deseasonalized | | -0.001 | -0.016-0.013 | 0.849 |
| **Baseline 25 (OH)D deseasonalized*time since baseline (per 10 nmol/L per year)** | | **-0.004** | **-0.008--0.000** | **0.041** |
| Baseline 25 (OH)D deseasonalized*MS phenotype at baseline (ref. = RRMS)(per 10 nmol/L) | | 0 |  |  |
|  | SPMS | 0.009 | -0.035-0.053 | 0.532 |
|  | PPMS | 0.009 | -0.035-0.053 | 0.689 |
| Baseline 25 (OH)D deseasonalized*MS phenotype at baseline (ref. = RRMS)*time since baseline (per 10 nmol/L per year) | |  |  |  |
|  | SPMS | 0.021 | -0.011-0.001 | 0.198 |
|  | PPMS | 0.013 | -0.039-0.066 | 0.621 |
| MS phenotype at baseline (ref. = RRMS) | |  |  |  |
|  | SPMS | -0.087 | -0.274-0.101 | 0.363 |
|  | PPMS | -0.059 | -0.323-0.206 | 0.664 |
| **Time since baseline (years)** | | **0.448** | **0.235-0.661** | **<0.001** |
| Age at baseline (years) | | 0.000 | -0.0031-0.0038 | 0.838 |
| Duration of disease (years) | | 0.003 | -0.002-0.008 | 0.288 |
| **Sex (ref. = female)** | | **0.092** | **0.001-0.183** | **0.046** |
| **EDSS baseline** | | **1.012** | **0.989-1.035** | **<0.001** |
| **EDSS baseline^2^ (centered around mean of 3.8)** | | **0.011** | **0.003-0.018** | **0.004** |
| Relapse rate 3 years pre-baseline | | 0.016 | -0.008-0.040 | 0.186 |
| MS phenotype at baseline (ref. = RRMS)*time since baseline (years) | |  |  |  |
|  | SPMS | 0.128 | -0.076-0.331 | 0.218 |
|  | PPMS | 0.098 | -0.210-0.405 | 0.534 |
| **Age at baseline (years)*time since baseline (years)** | | **0.005** | **0.002-0.009** | **0.006** |
| **EDSS baseline*time since baseline (years)** | | **-0.130** | **-0.154--0.105** | **<0.001** |
| **Relapse rate 3 years pre-baseline*time since baseline (years)** | | **-0.030** | **-0.057--0.003** | **0.029** |

*Corrected for duration of disease (years)*sex B -0.010, p=0.027*
